# Decreased Peripheral BDNF Levels and Cognitive Impairment in Late-Life Schizophrenia

**DOI:** 10.3389/fpsyt.2021.641278

**Published:** 2021-06-22

**Authors:** Lijuan Huo, Zhiwei Zheng, Xiaobing Lu, Fengchun Wu, Yuping Ning, Xiang Yang Zhang

**Affiliations:** ^1^Department of Psychiatry, Affiliated Brain Hospital of Guangzhou Medical University (Guangzhou Huiai Hospital), Guangzhou, China; ^2^Guangdong Engineering Technology Research Center for Translational Medicine of Mental Disorders, Guangzhou, China; ^3^CAS Key Laboratory of Mental Health, Institute of Psychology, Chinese Academy of Sciences, Beijing, China; ^4^The First School of Clinical Medicine, Southern Medical University, Guangzhou, China

**Keywords:** brain-derived neurotrophic factor, late-life schizophrenia, serum, cognitive deficits, attention

## Abstract

**Objectives:** There are relatively few studies on mechanisms of cognitive deficits in late-life schizophrenia (LLS). Brain-derived neurotrophic factor (BDNF), as an important neuroplastic molecule, has been reported to be involved in neurocognitive impairment in schizophrenia. This study aimed to examine whether peripheral BDNF levels were associated with cognitive deficits in LLS, which has not been explored yet.

**Methods:** Forty-eight LLS patients and 45 age-matched elderly controls were recruited. We measured all participants on the Repeatable Battery for the Assessment of Neuropsychological Status (RBANS) for cognition and serum BDNF levels. Psychopathological symptoms in patients were assessed by the Positive and Negative Syndrome Scale (PANSS).

**Results:** The levels of BDNF in LLS patients were significantly lower than those in healthy controls (8.80 ± 2.30 vs. 12.63 ± 5.08 ng/ml, *p* < 0.001). The cognitive performance of LLS patients was worse than that of the controls on RBANS total score and scores of immediate memory, attention, language, and delayed memory (all *p* ≤ 0.005). BDNF was positively associated with attention in LLS patients (*r* = 0.338, *p* = 0.019).

**Conclusion:** Our findings suggest that older patients with schizophrenia exhibit lower BDNF levels and more cognitive deficits than older controls, supporting the accelerated aging hypothesis of schizophrenia. Moreover, decreased BDNF is related to attention deficits, indicating that BDNF might be a candidate biomarker of cognitive impairments in LLS patients.

## Introduction

As the aging of the population has become a global trend, the number of older people with mental illness has increased significantly. Notably, late-life schizophrenia (LLS) accounts for the largest proportion of care expenditures for elderly patients with mental illness or other non-psychiatric disorders (including dementia), making it a major public health problem (1, 2). Nevertheless, the aging problem of schizophrenia has been ignored, especially on cognitive impairment (the core features of both schizophrenia and aging), and its pathophysiological mechanisms.

Cognitive deficits have been recognized as a core feature of schizophrenia for the past few decades, and research has surged in the last decade. Patients with schizophrenia suffer various degrees of cognitive impairment in a range of domains, such as processing speed, verbal memory, working memory, attention, executive functions, and visual memory (3, 4). Extensive cognitive impairments have seriously affected social ability, vocational rehabilitation, and independent life, leading to a poor prognosis for schizophrenia (5–7). Throughout the lifespan, cognition strongly predicts the functional capacity of schizophrenia independent of age (8). Concerning senile schizophrenia, significant cognitive impairments were generally reported compared with elderly healthy controls (9). Several meta-analyses showed moderate to large effect sizes across multiple cognitive domains (9–11), of which executive function, verbal fluency, and memory impairments were the most consistent. Although the cognitive decline in LLS patients has been well-documented, its underlying etiology and pathogenesis are still inconclusive.

Not yet been investigated, brain-derived neurotrophic factor (BDNF) may be involved in cognitive impairments of LLS. As a major member in the neurotrophin, BDNF plays a critical role not only in neurodevelopment and neuroprotection but also in synaptic plasticity, learning, and a variety of cognitive functions (12). Substantial evidence in animal studies showed that the up-regulation of BDNF signaling pathway or endogenous BDNF levels promoted neural development and memory capability (13, 14), while inhibition of BDNF signaling interfered with learning and long-term memory formation (15). It is consistent with clinical findings in human studies. Decreased peripheral BDNF levels have been widely demonstrated in both medicated and drug-naive first-episode patients with schizophrenia, potentially contributing to their cognitive impairments, revealed by recent meta-analyses (16–19). Lower BDNF levels predicted worse performance in many cognitive measurements, including attention, perceptual-motor skills, processing speed, and memory. Our study found that higher levels of BDNF corresponded to better performance on immediate memory (20). Based on findings in animal studies and adult schizophrenia, it is speculated that in LLS patients, cognitive performance is at least partially associated with peripheral BDNF levels. Yet so far, the relationship between peripheral BDNF levels and cognition in patients with LLS has not been explored (21), either which specific domains of cognitive deficits would be correlated with altered BDNF levels if any.

In this study, we examined serum BDNF levels and cognitive function in LLS patients to determine (1) whether the cognitive performance of LLS patients would be worse than that of healthy elderly; (2) whether the levels of BDNF in LLS patients would be lower than that of healthy elderly; and (3) whether there would be a correlation between BDNF and neurocognitive function of LLS patients.

## Methods

### Participants

Inpatients with geriatric schizophrenia were recruited from two public psychiatric hospitals in China, including Hui-Long-Guan hospital in Beijing and Rong-Jun hospital in Hebei province. All patients met the DSM-IV criteria for schizophrenia and had no other psychiatric disorders. Their diagnosis was confirmed by two independent experienced psychiatrists according to the Structured Clinical Interview for DSM-IV (SCID). A total of 48 chronic patients over 60 years of age (average age: 63.77 ± 2.94 years) were enrolled, of which 38 were men and 10 women. The average age of the first onset was 26.85 ± 6.07 years. Before participating in this study, all patients had been receiving stable antipsychotic medication for at least 6 months, with an average dose (in chlorpromazine equivalents) of 325.25 ± 159.59 mg/day. Antipsychotic drugs included clozapine (*n* = 17), risperidone (*n* = 17), perphenazine (*n* = 5), pipotiazine (*n* = 3), haloperidol (*n* = 2), sulpiride (*n* = 2), chlorpromazine (*n* = 1), and aripiprazole (*n* = 1). The average duration of treatment with the current medication was 42.72 ± 44.53 months.

Forty-five age-matched healthy controls were recruited from the local community in Beijing and they aged over 60 years, with an average age of 63.77 ± 2.94 years, including 27 men and 18 women. Current mental and physical conditions were assessed through structured clinical interviews according to the DSM-IV by psychiatrists. None of them suffered from mental disorders or neurodegenerative diseases.

A detailed demographic questionnaire was conducted for each participant, recording general information, smoking behavior, socio-demographic characteristics, and medical and psychiatric history. Medical records and collateral resources were also used to collect additional information. All participants were determined to be free of substance abuse or severe physical diseases. All of the participants signed a written informed consent form before participating in this study. The research protocol of this study was approved by the Ethics Committee of Beijing Hui-Long-Guan hospital.

### Clinical Symptoms and Cognitive Assessments

The Repeatable Battery for the Assessment of Neuropsychological Status (RBANS) (22) was used to assess the neurocognitive function of each participant. RBANS consists of five domains of cognition assessed with twelve subtests: immediate memory (List Learning and Story Memory), visuospatial/constructional ability (Figure copy and Line Orientation), language (Picture Naming and semantic fluency), attention (Forward Digit Span and Coding), and delayed memory (List Recall, List Recognition, Story Recall, and Figure Recall). Thus, five index scores and a total score were given to each participant according to the normative data.

The psychopathological symptoms of each patient were assessed by four independent psychiatrists by using the Positive and Negative Syndrome Scale (PANSS) (23), including a total score and scores of three subscales: positive symptoms, negative symptoms, and general psychopathology. To ensure the consistency of the rating scores, the psychiatrists attended the same training course in the use of PANSS. For repeated evaluation of the PANSS total score, the inter-rater correlation coefficient was >0.8.

### Serum BDNF Measures

Venous blood (10 ml) was collected into a non-anticoagulant tube between 7 and 9 a.m. after overnight fasting. After coagulation at room temperature, the serum was separated by centrifugation at 3,000 rpm for 10 min and then stored at −70 °C until assay.

The serum BDNF levels were measured within 1 month by sandwich enzyme-linked immunosorbent assay (ELISA) using a commercially available kit, according to the standard protocol (RayBio^®^ Human BDNF ELISA kit). Standard 96-well-plates were coated with the mouse monoclonal anti-BDNF immunoglobulin and incubated overnight. After washing, the samples and standards (concentration 0.1–256 ng/well) were incubated overnight. The plates were then washed three times with washing buffer, followed by incubation with chick anti-BDNF overnight. After three washes, a 1:1,000 dilution of peroxidase-labeled anti-chick antibody was added. After further washing, the reaction was developed at room temperature with tetramethylbenzidine (TMB) and stopped with phosphoric acid. Absorbencies were measured by a microtiter plate reader (absorbency at 450 nm).

Each evaluated parameter was assayed in duplicate for all samples. All samples were assayed by a technician who was blind to the clinical status of each subject. The identity of all subjects was indicated by a code number maintained by the investigator until all biochemical analyses were completed. Inter- and intra-assay variation coefficients were 8 and 5%, respectively.

### Statistical Analysis

The demographic variables of LLS and healthy elderly groups were compared with student's *t*-tests (continuous variables) and chi-squared tests (categorical variables). The levels of BDNF showed a normal distribution by Kolmogorov–Smirnov one-sample test (*p* > 0.05). We used analysis of covariance (ANCOVA) to compare the differences in BDNF and cognitive performance on RBANS between LLS patients and elderly controls, with demographic variables that showing significant between-group differences (i.e., sex, education, marital status) as covariates. The relationship between variables was examined by Pearson's correlation coefficients. Bonferroni correction was applied for multiple testing. Further, we used multivariate regression analyses (enter regression model) to examine the relationship between cognitive function (RBANS) and BDNF while controlling the demographic and clinical variables including antipsychotic treatment (type, dose, and duration of treatment) and clinical symptoms on PANSS.

We utilized SPSS (version 24.0) to carry out all statistical analyses and set the significance level α at 0.05 and *p*-value to two-tailed.

## Results

The demographic characteristics of LLS patients and healthy elderly controls are summarized in Table 1. There were significant differences in sex, education, and marital status (all *p* < 0.05) between the two groups, but without significant differences in age, BMI, and smoking status. In the following analyses, sex, education, and marital status were adjusted.

**Table 1:** Characteristics of late-life schizophrenia (LLS) and healthy older adults.

	**LLS (*n* = 48)**	**Healthy older controls (*n* = 45)**	***t*** **or** ***χ***^**2**^	***p***
Age (years)	63.77 ± 2.94	64.56 ± 3.24	−1.23	0.224
Sex (male %)	79.2%	60%	4.06	0.044
Education (years)	9.53 ± 2.94	7.48 ± 3.60	3.02	0.003
BMI (kg/m^2^)	24.86 ± 5.88	25.81 ± 3.31	−0.716	0.480
Marital status			27.77	<0.001
Single	41.7%	0%		
Married	29.2%	73.3%		
Divorced or widowed	29.2%	25.7%		
Smokers	56.3%	55.6%	0.005	0.946
Age of onset (years)	26.85 ± 6.07	–		
Antipsychotic		–		
Atypical	81.3%	–		
Typical	18.8%	–		
Antipsychotic dose (mg/day) (chlorpromazine equivalents)	325.25 ± 159.59	–		
**PANSS**				
Total score	66.02 ± 11.27	–		
Positive subscore	13.06 ± 5.62	–		
Negative subscore	24.34 ± 6.68	–		
General psychopathology subscore	28.34 ± 4.66	–		

*PANSS, the positive and negative syndrome scale*.

### BDNF in LLS and Healthy Older Controls

Serum BDNF levels were significantly lower in LLS patients than that in healthy elderly (8.80 ± 2.30 vs. 12.63 ± 5.08 ng/ml, *F* = 22.30, *df* = 1, *p* < 0.001). The difference in BDNF levels between the two groups remained significant after adjusting for sex, marital status, and education as covariates (*F* = 21.72, *df* = 1, *p* < 0.001).

BDNF serum levels were not associated with any demographic variables in either the control group or the LLS group (all *p* > 0.05). In the LLS patient group, BDNF levels were not related to the age of onset, duration of disease, and type and dosage of antipsychotic treatment (all *p* > 0.05). Correlation analysis revealed that there were no significant correlations between serum BDNF levels and PANSS positive symptom, negative symptom, and general psychopathology subscale and total scores in LLS patients (all *p* > 0.05).

### Cognitive Functions of LLS and Healthy Older Controls

Table 2 displays the RBANS performance of LLS patients and healthy controls. Expect for visuospatial/constructive domain, LLS patients exhibited poorer cognitive performance in terms of immediate memory, attention, language, delayed memory, and total scores of RBANS compared to healthy elderly controls. After adjusting for sex, marital status, and education, these differences remained significant (all *p* < 0.05). In the LLS group, PANSS negative symptom was negatively associated with all RBANS total and domain scores (all *p* < 0.05), except for the visuospatial/constructional index.

**Table 2:** Total and each index scores on the RBANS in late-life schizophrenia (LLS) vs. healthy older controls.

	**LLS (*n* = 48)**	**Healthy older controls (*n* = 45)**	***F***	***p***	Adjusted F^*^	Adjusted p^*^
Immediate memory	56.02 ± 13.17	73.78 ± 16.49	33.13	<.001	39.81	<0.001
Attention	73.75 ± 14.93	84.44 ± 20.29	8.46	0.005	12.21	0.001
Language	82.15 ± 14.73	92.69 ± 11.63	14.54	<0.001	23.05	<0.001
Visuospatial	79.54 ± 20.48	75.98 ± 12.76	1.00	0.320	0.01	0.93
Delayed memory	66.04 ± 19.31	87.11 ± 14.81	34.52	<0.001	42.49	<0.001
Total scale	65.08 ± 13.20	77.64 ± 13.54	20.53	<0.001	24.85	<0.001

* *Adjusted values were calculated with sex, marital status, and education as covariates. RBANS, the Repeatable Battery for the Assessment of Neuropsychological Status*.

### Correlation Between BDNF and Cognitive Performance

**Figure 1:**
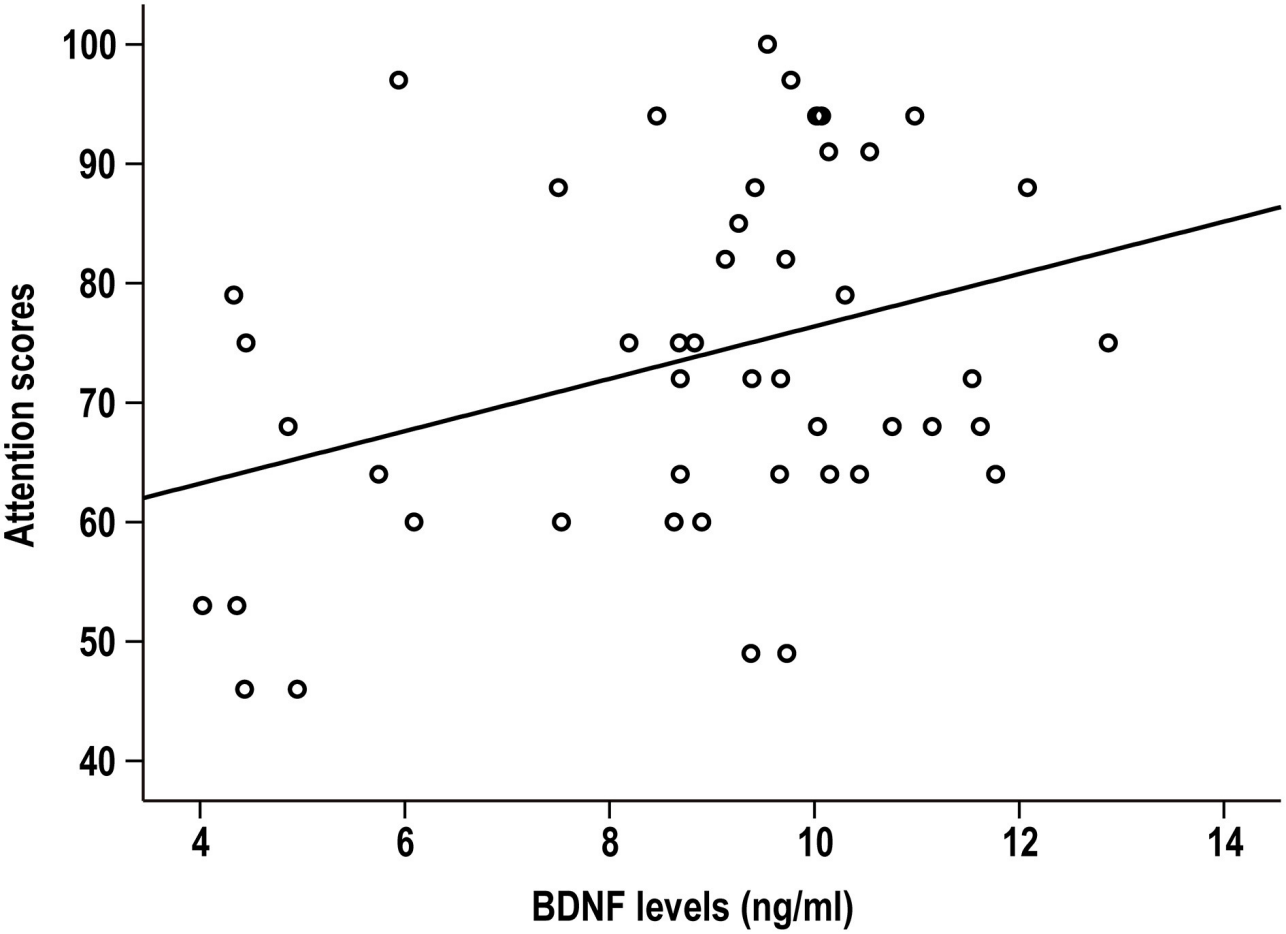
Serum BDNF was positively associated with the attention index in patients with late-life schizophrenia (*r* = 0.338, *p* = 0.019).

In LLS patients, BDNF levels were positively correlated with attention index (*r* = 0.338, *p* = 0.019, Figure 1). Partial correlation analysis further confirmed this significant association by controlling for sex, marital status, and education (*r* = 0.371, *p* = 0.013). However, the association did not survive Bonferroni correction (*p* > 0.05/5). There was no significant association between BDNF and other cognitive indexes (all *p* > 0.05). Serum BDNF levels were not associated with any RBANS scores in healthy elderly controls (all *p* > 0.05).

Further, multiple linear regression analysis was conducted to explain the contribution of BDNF, demographic and clinical variables, and antipsychotic treatment to the attention index of LLS patients. The results showed that BDNF levels (*β* = 0.27, *t* = 2.10, *p* = 0.042), antipsychotic type (*β* = 0.27, *t* = 2.08, *p* = 0.045), and years of education (*β* = 0.39, *t* = 3.10, *p* = 0.004) were independent contributors to attention index. Together they could explain the 42% variation in attention index. BDNF was not correlated to other RBANS domain and total scores.

## Discussion

To our best knowledge, this is the first study to explore the peripheral BDNF level and its relationship with cognitive function in late-stage schizophrenia. There were three main findings in this study: (1) compared with age-matched healthy elderly, serum BDNF levels were significantly lower in LLS patients; (2) LLS patients displayed extensive cognitive impairments in all RBANS total and domain scores except for visuospatial/constructional domain; (3) BDNF levels were associated with attention in LLS patients.

We found that LLS patients had severe and pervasive cognitive deficits compared with healthy elderly controls. This is consistent with most previous studies evaluating the various cognitive performance of LLS patients (9, 10). Also, study in our lab (24) found cognitive impairments in younger populations with schizophrenia in global cognition and multiple domains, i.e., language, executive function, and memory, measured by the MATRICS Consensus Cognitive Battery (MCCB), suggesting that schizophrenia have widespread cognitive impairments without categorical differences in both adulthood and late-life stages.

Our finding of lower BDNF serum levels in LLS patients compared with the aged-matched cohort, extended previous findings in younger and middle-aged schizophrenia (16, 25–29). Regarding BDNF levels of patients with LLS, a few studies with rare samples assayed BDNF in post-mortem brain tissue (30–33). These studies demonstrated that BDNF levels and protein levels of calbindin-D and TrkB receptors, which were up-regulated by BDNF, were significantly reduced in the hippocampus or pre-frontal cortex. This study examining the levels of BDNF in the blood *in vivo* for geriatric patients with schizophrenia indicated a reduction of blood BDNF persists with age and also provided evidence for the association between blood and brain BDNF levels. Taking together evidence *in vitro* and post-mortem studies, BDNF may be involved in the pathophysiology of schizophrenia at all ages.

Decreased BDNF and cognition in LLS patients verified the accelerated aging hypothesis of schizophrenia (34), which suggests that schizophrenia is a syndrome of accelerated aging, because patients exhibit signs of deterioration, including physical illness, cognitive decline, metabolic problems, and shortened life expectancy, similar to the aging process but at an accelerated rate. Parallel with this hypothesis, a recent study using neuroimaging technology and machine learning demonstrated that mental illness accelerated normal brain maturation, resulting in an older estimated brain age in schizophrenia patients than in healthy controls (35). Moreover, at the behavioral level, cognitive performance and daily functional skills of patients with schizophrenia were as poor as those of healthy individuals who were three decades older (36). Consistently, our results show that BDNF and cognition were lower in LLS patients than those of age-matched healthy elderly, which may be the sign of premature aging. Nevertheless, the underlying neurobiological connections and interactions between aging and schizophrenia are uncertain and need further investigation. Further longitudinal studies on patients of different ages can provide more detailed explanations.

Another important finding is that cognitive performance (i.e., attention), rather than psychotic symptoms, was positively associated with serum BDNF levels in LLS patients. As a molecular marker of neuroplasticity, BDNF plays a key role in regulating synaptic structure and function. In animal models, as the essential mechanisms of learning and memory, long-term potentiation (LTP) was impaired in BDNF-knockout mice (37, 38) and could be rescued by acute infusion of BDNF (14). In adult mice, hippocampus-specific deletions of the BDNF gene resulted in impaired object recognition and spatial learning in the Morris water maze (39). It is worthy of mentioning that although in our study, we assessed BDNF levels in the blood rather than in the central nervous system, the BDNF concentrations in the blood reflected brain-tissue BDNF levels across species (40–42). Indeed, it is generally recognized that blood BDNF levels were associated with cognitive function as well. For instance, BDNF serum levels were significantly reduced in almost all cognitively-impaired groups, such as mild cognitive impairment (43), Alzheimer's disease (44), and Parkinson's disease (45). Similarly, our results together with previous studies, verified that in patients with schizophrenia, cognitive impairments were always accompanied by lower blood BDNF levels (19, 20). Besides, some studies proposed the neurotrophin hypothesis of schizophrenia, which suggested that changes in neurotrophic factors resulted in disturbed processes of neuroplasticity and neural maldevelopment, thereby contributing to the pathogenesis of schizophrenia (46). However, we did not find a significant correlation between BDNF levels and the severity of positive or negative symptoms, which were in agreement with some previous studies (17), suggesting that low BDNF levels may have a small and insignificant relationship with the psychopathology of schizophrenia, or it may be just a pathological epiphenomenon of schizophrenia.

Interestingly, we found that decreased BDNF levels were only associated with greater severity of attention deficits. One possible explanation is that attention is more susceptible to lower BDNF interference than other cognitive domains. Several lines of evidence from patients with attention deficit hyperactivity disorder (ADHD) indicated that decreased midbrain BDNF activity might be implicated in the pathogenesis of attention deficits, while pharmacotherapy for ADHD upregulated BDNF expression in the brain (47, 48). Other domains of cognitive function, such as memory, were affected by more factors in addition to BDNF, such as up-regulated inflammatory factors, Aβ amyloid deposition, and microtubule-associated tau protein pathology. In addition, it is important to consider that in RBANS, attention ability was measured by the digit span test, which was also a traditional psychology test for working memory (24, 49). In this task, information was required to be stored in immediate memory and reproduced later. Thus, we speculated that serum BDNF levels were related to working memory deficits in schizophrenia.

This study has several limitations. First, a long period of illness may lead to hospitalization, sedentary lifestyle, chronic antipsychotic treatment, comorbid depression, and anxiety. These confounding factors may partly account for the differences in BDNF and cognitive function between the patient group and the control group. Unfortunately, we did not collect all the confounders to control for their possible effects on BDNF levels and cognition. Therefore, caution should be taken in interpreting these results. Second, we did not conduct cognitive screening by neuropsychological tests. Dementia was ruled out only by the structured clinical review and psychiatric evaluation based on DSM-IV. As a result, LLS may have mild cognitive impairment. Third, due to the cross-sectional design, this study could not reveal the causal relationship between decreased BDNF and cognitive impairment in geriatric schizophrenia patients. Fourth, our results were preliminary with relatively small sample size, and further validation will be conducted in an expanded sample of LLS patients to draw a firm conclusion. Fifth, there are some limitations regarding the assays of BDNF. Although platelets are the major source of peripheral BDNF, we did not analyze the BDNF level in the framework of platelet counts. In the future, it would be interesting to investigate the relationship between BDNF levels in platelets and cognitive functions. In addition, there may be some differences in performance of commercial BDNF assays, as recently demonstrated by Polacchini et al. (50). As a result, although we have conducted the BDNF assays strictly following the manual, it should be cautious to compare our results with the published blood-derived BDNF data.

In conclusion, our study is the first step in characterizing reduced peripheral BDNF levels and large-scale cognitive deficits in older schizophrenia, supporting the accelerated aging hypothesis of schizophrenia. Also, we revealed that decreased BDNF levels are linked to attention deficits but not psychotic symptoms of LLS patients, indicating that BDNF might be a reliable biomarker of cognitive function. Further biological and longitudinal studies with larger samples are needed to elucidate the underlying mechanism underlying the association between low BDNF serum levels and cognitive deficits in LLS patients, and how aging and schizophrenia interact in cognitive changes.

## Data Availability Statement

The raw data supporting the conclusions of this article will be made available by the authors, without undue reservation.

## Ethics Statement

The studies involving human participants were reviewed and approved by the Ethics Committee of Beijing Hui-Long-Guan hospital. The patients/participants provided their written informed consent to participate in this study.

## Author Contributions

LH, YN, and XZ were responsible for study design and data analysis. FW and XL were responsible for data acquirement. LH, ZZ, YN, and XZ drafted the manuscript. All the authors critically reviewed the manuscript and gave final approval for its publication.

## Conflict of Interest

The authors declare that the research was conducted in the absence of any commercial or financial relationships that could be construed as a potential conflict of interest.
